# Physical Contests for Females in the Japanese Beetle, *Popillia japonica*

**DOI:** 10.1673/031.007.3401

**Published:** 2007-05-22

**Authors:** Kipp C. Kruse, Paul V. Switzer

**Affiliations:** Department of Biological Sciences, Eastern Illinois University, Charleston, Illinois 61920

**Keywords:** Coleoptera, scramble competition, fighting behavior, body size, prior residency advantage

## Abstract

We conducted field observations of physical competition for mates, in which a single male attempts to usurp a female from another male, in male Japanese beetles, *Popillia japonica* Newman (Coleoptera; Scarabaeidae). Physical contests for mates were relatively rare, but when they occurred the challenger male was able to successfully takeover females by dislodging the previously paired resident male in only 18% of contests, suggesting that a substantial prior residency advantage exists in this species. Challenger males that were successful in takeover attempts were significantly larger than the resident male. In contrast, no size pattern was found between intruding males and residents in unsuccessful takeover attempts. The frequency of contests for existing pairs was examined throughout the day. Pair frequency was greatest in early morning and in the evening but contest frequency was highest during the middle of the day. Contest frequency was negatively related to beetle density but not related to temperature. Overall, physical contests for mates appear to be an important part of the mating behavior in Japanese beetles. The frequency of the contests relates to the time of day and social conditions and contest outcome is related to prior residency and the size of the intruding male relative to the paired male.

## Introduction

In many insects, males compete directly with other males for access to females (Thornhill and Alcock 1983). Observations of insect males being dislodged from females by other males during mating are common (Thornhill and Alcock 1983) and have been observed in a variety of species (McLain and Boromisa 1987; Otronen 1989; Johnson 1982; Sugiyama et al. 1992; Enders 1995; Sigurjonsdottir and Snorrason 1995). In insects with direct, physical competition, the outcome of the contest is frequently related to male size, with larger males tending to win (Thornhill and Alcock 1983). In addition, prior residency also may be related to contest outcome, with the resident seeming to win a higher percentage of interactions than would be expected when considering other factors such as size (e.g., Davies 1978; Otronen 1984; Thornhill 1984).

Social conditions, and time of day, may affect contest behavior. For example, studies of territorial insects commonly find that an increase in density often increases the number of competitive interactions (Pezalla 1979; Koenig and Albano 1985). In addition, contests often occur more often at some times of day than others. For example, in the territorial dragonfly *Paltothemis lineatipes*, contests were more common in the morning, prior to female arrival (Alcock 1987). Temporal differences in contest frequency can be related to physical conditions such as temperature (Pezalla 1979) and, in some cases, may ultimately be related to an increase in the benefit to fighting. For instance, female availability or receptivity may vary temporally. Consequently, fighting among males may correspond to female availability in such a way as to provide males access to females at the times when females are most available (Koenig and Albano 1985; Alcock 1987).

In this study, we examined contest behavior in Japanese beetles, *Popillia japonica* Newman (Coleoptera; Scarabaeidae). The Japanese beetle is a common, introduced species throughout the eastern part of the USA and is a horticultural and agricultural pest in both the larval and adult stages (Fleming 1972; Potter and Held 2002). Virgin females emit a sex pheromone (Ladd 1970; Tumlinson et al. 1977), but they stop producing this pheromone after their first mating (Ladd 1970), even though they may copulate and lay eggs repeatedly during their 1–2 month adult lifespan (Fleming 1972; Van Timmeren et al. 2000). Males and females aggregate on food plants in response to plant kairomones (Loughrin *et al* 1995). Japanese beetles are promiscuous (Fleming 1972; Barrows and Gordh 1978; Potter and Held 2002), with both sexes capable of copulating with multiple partners on the food plants. Male mating behavior can best be described as “scramble competition” (Thornhill and Alcock 1983; KCK and PVS unpublished observations) and after mating, males may continue to ride on the backs of females for hours, guarding them from other males (Fleming 1972; Barrows and Gordh 1978; Saeki et al. 2005a, b). Although the level of aggregation varies widely (Switzer et al. 2001), Japanese beetles often occur in very high densities (Fleming 1972; Switzer et al. 2004).

Japanese beetle mating behavior seems to have both a competitive and temporal component. Single males may attempt to takeover females from paired males in the field (Fleming 1972; Thornhill and Alcock 1983; Potter and Held 2002). Furthermore, time of day and temperature appear to affect some aspects of Japanese beetle mating behavior. Switzer et al. 2001 found that Japanese beetles paired, and thus most likely copulated, more frequently in the morning and evening and at lower temperatures. The sex ratio on the food plant, although male-biased, did not change consistently over the course of a day and unpaired females and males existed throughout the day (Switzer et al. 2001). Male-male pairs were more common in the afternoon (Switzer et al. 2004).

Relatively little is known about the mating dynamics of the Japanese beetle. It is not known to what extent physical competition between males is involved in mate acquisition. Nor is it known whether this physical competition was associated with factors such as pair frequency, density, temperature that may correlate with the scarcity of single females or difficulty in searching for these females; conditions that may increase the net benefit to fighting relative to other options (Thornhill and Alcock 1983). As in many insects, in Japanese beetles the last male to copulate with a female fertilizes the majority of the eggs (Ladd 1966; Simmons 2001) so the outcome of a fight has important fitness consequences. The primary objectives of this study were two-fold: first, to observe naturally occurring contests in the field to determine factors affecting the outcome of these contests, and second, to determine the number of contests over the course of a day to test whether contest frequency is associated with pair frequency, density, or temperature.

## Materials and Methods

We observed Japanese beetles in east-central Illinois (Coles and Cumberland County) over a 4 yr time span. For beetles that were collected, sex was determined by differences in foreleg morphology (Smith and Hadley 1926) and body size was determined by recording maximum body width using a dissection scope with an ocular micrometer. Maximum body width occurs at the anterior portion of the elytra and is a good predictor of overall body size (Van Timmeren et al. 2000).

### Focal observations of fights

In July 2000 and 2002, focal observations were made of naturally-occurring male-male interactions in soybean fields. A ‘fight’ was defined as when one or more males were in active, physical contact with a male that was mounted to a female; simple, brief contact between males was not recorded as a fight. Data were analyzed only on fights between one intruding male and one resident male.

Most observations were made between 1400 and 1700 hr. Because fights are relatively rare in the field, observations were made on fights that had already started; therefore, the beginning of a fight was rarely included in the focals. For each focal, the duration of the fight was recorded to the nearest second, and the fate of the loser (crawled away, flew away, stayed within 1 cm of the pair) was noted. Fights were considered to be over if the loser left the pair and did not reinitiate contact. Because the beginning of the fight was not observed, the durations of the fights were reported for descriptive purposes and we did not include analyses on factors affecting these durations. Following the fight, an attempt was made to capture all the individuals involved. Although 104 fights were observed, sample sizes differ for some of the analyses because not all individuals were caught for all fights.

### Temporal variation in fights

For 10 days in 2003 (between 31 July and 13 August), we recorded the frequency of fights at 5 sampling time periods (0700, 1000, 1300, 1600, and 1900 hr). At each time period, we sampled 300 pairs in the same soybean field by walking in one direction down the edge of the field, carefully searching for all single and paired beetles within 2 adjacent rows, and recording, for each pair found, whether it was alone or in a “fight” with a third individual. To distinguish fights from brief interactions, we defined a fight as an interaction between an intruding male and a pair that lasted for more than 5 s. To estimate pair frequency, we also counted the number of single individuals observed in the same area for which the first 100 pairs were found, added those individuals to the number of individuals in pairs, and calculated the number of pairs per total number of beetles in the area. We recorded, to the nearest 1 m, the length of the sampling transect required to find 100 pairs; this length provided us with an estimate of density (i.e. we added the number of single and paired beetles and divided by the length of the sampling transect to get individuals/m). Temperature was recorded at the beginning of each sampling period. Samples were not taken during rain; this resulted in sample sizes differing for some time periods. We analyzed these data by conducting an ANCOVA with the number of fights as the dependent variable and time period, density, pair frequency, and temperature as independent variables.

We analyzed all data with either JMP 3.1 (SAS Institute 1995) or Statview 4.5 (Abacus Concepts 1994). Means are presented ± SE. Nonparametric analyses were used when data did not conform to parametric assumptions. P-values for non-parametric statistics take tied values into account when appropriate.

## Results

### Fight observations

Male fighting behavior typically consisted of the intruding male trying to pry loose the mounted male (hereafter the resident) by inserting his head between the resident and female and pushing with his legs to gain leverage. The resident also pushed at the intruding male with his legs, attempting to keep the intruder off the female. If the intruding male loosened or partially displaced the resident, he then also used his legs to push the resident male completely off of the female. Often during contests males movement would cease and males would remain in position (e.g. one male partially inserted under another) for seconds to minutes. Males were never observed biting other males during these contests. Once usurped, the resident male usually attempted to reclaim the female by trying to pry loose the newly mounted intruder. Most intruding males (approximately 80%) were making their attempt from the front or behind the pair, as opposed to from the side or underneath. Generally, fights involved a single male attempting to dislodge a mounted male from a female; on some occasions, however, multiple males were involved.

**Figure 1.**
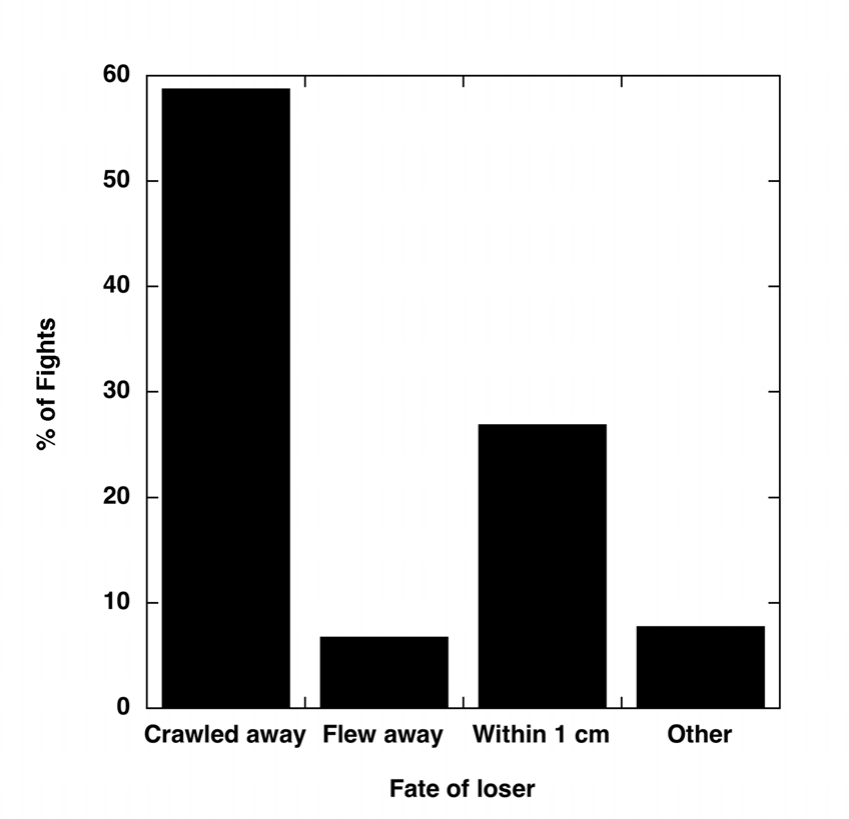
Behavior of the losing male Japanese beetle after the fight (N = 104 takeover attempts). “Other” includes those fights in which the fate of the loser was less clear because at least one additional male had joined the fight.

### Outcomes of Fights

The outcomes of 104 naturally-occurring fights in the field were observed. These fights lasted 437.6 ± 59.9 s on average (range: 13-3805 s, N = 104), with a skewed distribution (median = 218.5 s). Twenty-eight (27%) of the fights lasted less than 2 minutes, and 64 (61.5%) lasted less than 5 min. In 19 (18%) of these fights, the intruding male was successful in taking over the female. In 8/104 (7.7%), the female detached herself from both males (either as a result of falling off a leaf or crawling away). Thus, in approximately 74% of the fights, the resident male remained with the female. A male was never observed to regain the female after being displaced by another male. Losing males typically crawled away from the pair or stayed within 1 cm of the pair (Figure 1). Interestingly, in 5/104 (4.8%), the fight resulted in a homosexual pair forming between two males (i.e. one male was mounted on the other male and the female was separate), and in 4 additional fights, one male was observed probing the other male with his aedeagus at some point during the fight.

### Size and fight outcome

**Figure 2.**
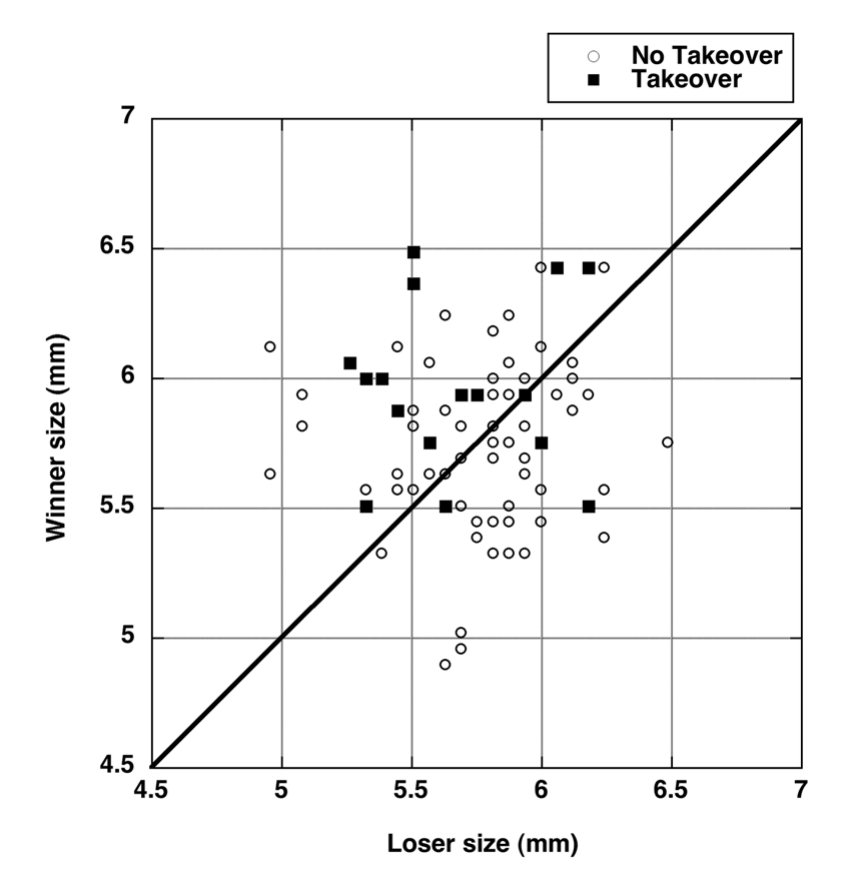
Comparison of sizes of Japanese beetle males involved in unsuccessful (N = 65) and successful (N = 16) takeover attempts. The line indicates resident and intruder beetles of equal size; therefore, points above the line represent cases in which the winners were larger than the loser.

Intruding males tended to be larger than residents, but this difference was not significant either when contests were pooled (Figure 2; df = 160, t = 1.65, P = 0.10) or when residents and intruders were compared within a given fight (mean difference (resident - intruder) = - 0.082 ± 0.048 mm, df = 80, paired t-test, t = 1.70, P = 0.09). Additionally, the size of the winner (regardless of whether it was resident or intruder) was not significantly different from the size of the loser (mean difference = 0.034 ± 0.049 mm, N = 81, paired t-test, t = 0.69, P = 0.49). However, when data were separated by whether the takeover attempt was successful, an interesting pattern emerged (Figure 2). Intruders were significantly larger than residents in 12/16 (75%) of the successful takeovers (mean difference (winner - loser) = 0.29 ± 0.11 mm, N = 16, paired t = 2.71, P = 0.016), but intruders were not consistently larger in unsuccessful takeover attempts (mean difference = -0.03 ± 0.05 mm, N = 65, paired t = 0.57, P = 0.57). Therefore, when a takeover attempt was successful, the intruding male was usually larger than the resident. However, just because the intruder was larger did not mean that he would necessarily be successful; in 33/62 (53%) of unsuccessful takeover attempts, the intruder was larger than the resident (Figure 2).

No other aspects of body size seemed to affect fight outcome. Neither female size (successful = 6.11 ± 0.13 mm, unsuccessful = 6.25 ± 0.04 mm; t = 1.4, df = 78, P = 0.16), paired male size (Figure 2; t = 0.71, df = 79, P = 0.48), or paired male to female size ratio (successful = 0.94 ± 0.02, N = 15, unsuccessful = 0.92 ± 0.008, N = 65, t = 0.97, df = 78, P = 0.33) were significantly different between successful and unsuccessful attempts.

**Figure 3.**
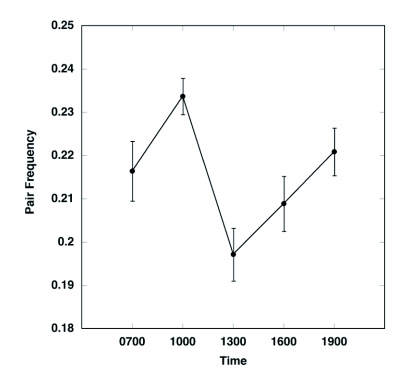
Mean frequency (± SE) of a) pairs (# pairs/individual) at 5 time periods for 10 days in 2003. All observations were made in the same soybean field. Observations were not made during rain, so the actual sample size for each time period differs (N = 10 days for 700, 1000, and 1300; N = 9 days for 1600 and 1900).

### Temporal pattern of fights

**Figure 4.**
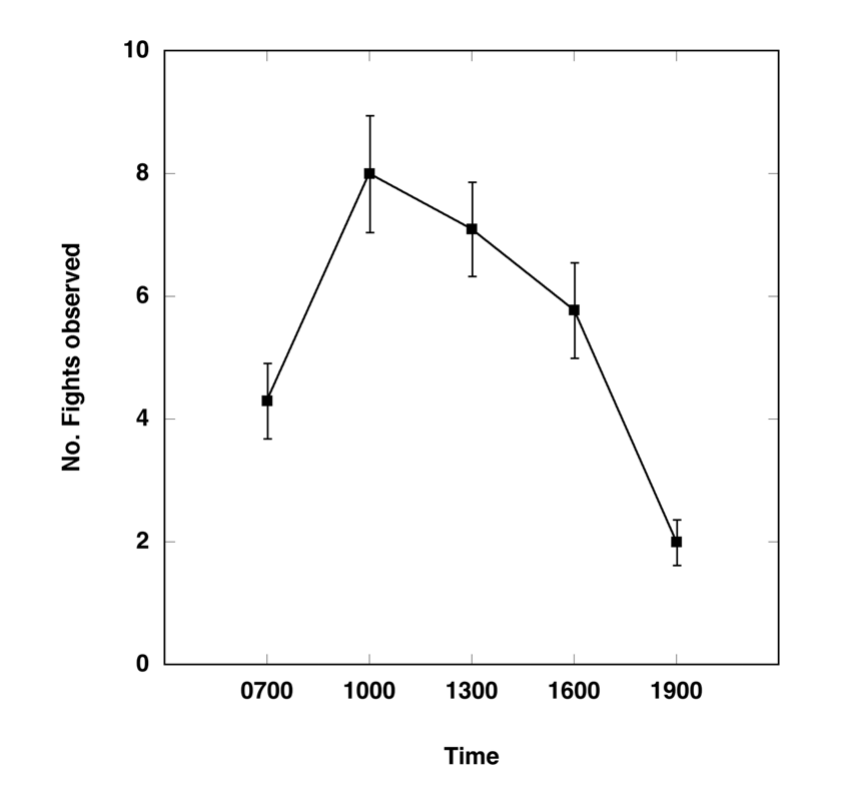
Mean frequency (± SE) of fights (per 300 pairs) at 5 time periods for 10 days in 2003. All observations were made in the same soybean field. Observations were not made during rain, so the actual sample size for each time period differs (N = 10 days for 700, 1000, and 1300; N = 9 days for 1600 and 1900).

**Figure 5.**
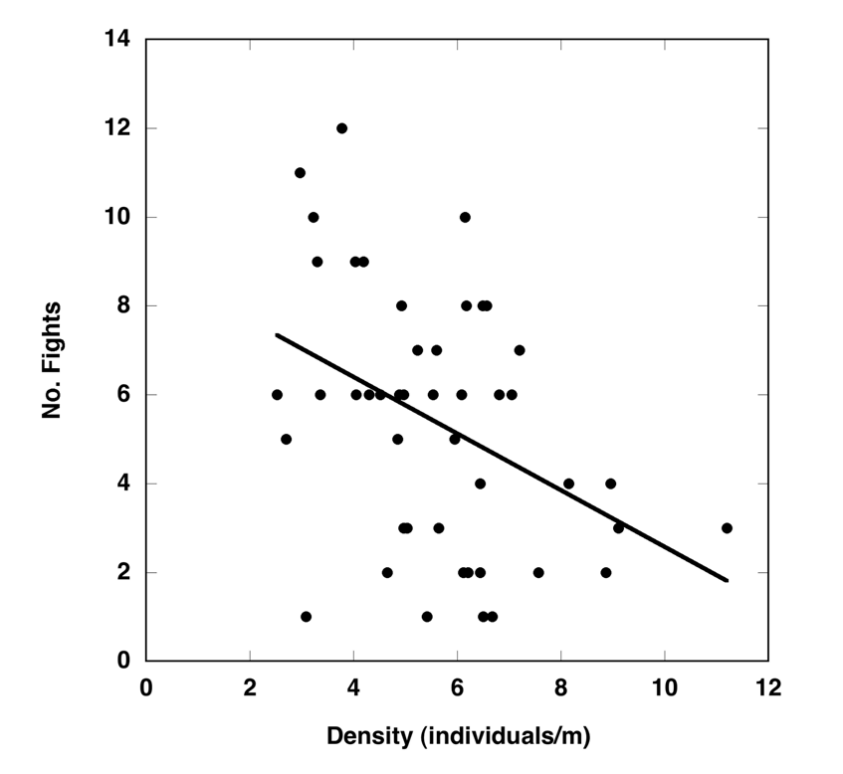
Relationship between the number of fights observed (per 300 pairs) and density of individuals for Japanese beetles observed in the same soybean field for 10 days in 2003. Relationship is pooled over all time periods and days; the line represents a simple linear fit to the data.

The frequency of pairs varied significantly throughout the day, with the lowest frequency in the afternoon and highest in the morning and evening (Figure 3; ANOVA; F 4,47 = 5.5, P = 0.001). At a given time, approximately 1–3% of the pairs experienced fights (Figure 4). Results of the ANCOVA (Whole model R^2^ = 0.63, F _7,44_ = 8.95, P < 0.0001) indicate that the frequency of fights varied consistently with time of day (F_4,44_ = 10.2, P < 0.0001) and density (Figure 5; F_1,44_ = 14.9, P = 0.0004) but not with temperature (F_1,44_ = 1.18, P = 0.28) or pair frequency (F_1,44_ = 0.17, P = 0.68); similar results were found when the density of single beetles rather than overall beetle density was included in the model. Interestingly, the fight frequency was highest at 1000 and 1300 and was negatively related to density.

## Discussion

### Fights and fight outcome

Although direct, physical competition for mates was not common in Japanese beetles, fights were consistently observed for a small percentage of pairs throughout our study. In these fights, the intruding male was successful in usurping the female in less than 20% of contests; overall, the paired male retained possession of the female in approximately 75% of contests. Therefore, paired, male Japanese beetles appear to have a substantial advantage in contests over females.

Such a residency advantage might be explained by the paired male's positional advantage, his higher fighting ability, the ‘value’ he attaches to the female, or some combination of these (Parker 1974, Parker and Rubenstein 1981, Austad 1983, Enquist and Leimar 1983). The most likely possibility seems to be that guarding males experience a positional/mechanical advantage by having a firm grasp of the female with their forelegs and middle legs and being able to push the intruding male with their hind legs (Fleming 1972, Barrows and Gordh 1978, KCK and PVS personal observation). This positional/mechanical advantage seems to be unrelated to the relative size of the paired male and female because no relationship was found between fight outcome and the relative sizes of the paired beetles. Fighting ability may also be related to energy levels (e.g. Marden and Waage 1990; Marden and Rollins 1994; Plaistow and Siva-Jothy 1996), but whether paired males have higher energy levels is unknown. In terms of resource value, the paired male may value the female more than the intruder in some cases (e.g. Austad 1983), but, given the last male advantage demonstrated in this species (Ladd 1966), why this would be true in Japanese beetles is unclear. One possibility, however, is that a paired male may have more information on when the female may leave to oviposit.

Although residency was found to be of primary importance in determining contest outcome, male size also played a role. In those cases with a successful takeover, the intruding male was significantly larger than the paired male. Body size is a frequent determinant of contests in insects and other taxa (Thornhill and Alcock 1983), including other beetles (McLain and Boromisa 1987; Enders et al 1998). In the case of Japanese beetles, large males may be able to overcome the suggested positional advantage of the paired male. However, relatively small males did attempt takeovers and relatively large males did fail in attempted takeovers, so fight initiation and outcome are not yet completely explained.

In this study, single contests were studied; it is not known how frequently males have to defend their females or whether the probability of winning each contest is independent of prior contests. In other insects, the amount of fighting a male has participated in and/or his energy reserves have a negative impact on his probability of winning future contests (Marden and Waage 1990; Switzer 2004); furthermore, the probability that the winner of a fight ultimately ends up fertilizing that female's eggs is not known. Consequently, by focusing on single contests we may not have a complete picture of the role of direct, male-male competition in the mating system of this species or on the fitness of individual males. Nevertheless, this study identifies the importance of both residency and body size in fight outcome, and, from the perspective of an intruding male, the probability of usurping the female should be consistent with these results.

### Temporal and social aspects of fight frequency

Pair frequency was found to be highest in the morning and evening and lowest in the middle of the day, a pattern also found previously (Switzer et al. 2001). Interestingly, fights for females had the opposite pattern, with fights being more frequent in the middle of the day and lowest in the morning and evening. Furthermore, fights were more common at low densities. These patterns cannot simply be explained by air temperature, because no significant, independent effect of temperature on fight frequency was found. It was also unlikely that these patterns were due to changes in population sex ratio, because an earlier study found no consistent changes in sex ratio over the course of the day (Switzer et al. 2001).

We suggest that one possible explanation for the temporal and density patterns relates to the availability of receptive females. If males can more readily find receptive or high quality females in the morning and the evening, and at higher densities, then males may be more likely to search for an unguarded female at these times than to pay the cost associated with a takeover attempt. In the middle of the day, single females may be either physically unavailable (i.e. occupying inconspicuous locations on the food plants), be behaviorally unavailable (i.e. physically resisting male mating attempts), be small in size, or have fewer eggs (Saeki et al. 2005c). Although these possibilities are still speculative at this point, future studies examining the spatial locations of females, female resistance to mating, and male mate-searching behavior would greatly help to understand the respective roles of male and female behavior in the fighting behavior of this species.
